# A Retrospective Study Comparing a Patient-specific Design Total Knee Arthroplasty With an Off-the-Shelf Design: Unexpected Catastrophic Failure Seen in the Early Patient-specific Design

**DOI:** 10.5435/JAAOSGlobal-D-19-00143

**Published:** 2019-11-04

**Authors:** Carlos J. Meheux, Kwan J. Park, Terry A. Clyburn

**Affiliations:** From the Houston Methodist Orthopedics & Sports Medicine, Outpatient Center, Houston, TX.

## Abstract

Patient-specific design (PSD) total knee arthroplasty implants are marketed to restore neutral mechanical-axis alignment (MAA) and provide better anatomic fit compared with standard off-the-shelf (OTS) total knee arthroplasty designs. The purpose was to compare the Knee Society scores, radiographic outcomes, and complications of PSD and OTS implants.

**Methods:**

Retrospective study analyzing PSD and OTS by a single surgeon. Implant design change in PSD occurred during the period of data collection leading to PSD-1 and PSD-2 subgroups. Radiographic data including MAA, femorotibial angle, coronal-tibial angle, tibial slope and patella-sulcus angle, and complications were analyzed. Minimum follow-up was 2 years or until revision, and patients completed Knee Society scores preoperatively and postoperatively at 3, 6, 12, 24 weeks, and final follow-up.

**Results:**

There were 136 patients (154 knees), average age (62.76 +/− 8.4 years), and follow-up (3.1 +/− 1.5 years). The groups included PSD-1 (77 knees), PSD-2 (36 knees), and OTS (41 knees). The PSD-2 group had better Knee Society function scores compared with PSD-1 and OTS at all timepoints except final follow-up. PSD-2 had significantly shorter hospital stay (*P* = 0.000012) and less hemoglobin drop (*P* = 0.032) compared with PSD-1 and OTS. No differences were observed in MAA (P = 0.349) or final range of motion (P = 0.629) between the 3 groups. PSD-2 had more normal femorotibial angle, coronal-tibial angle, and tibial slope compared with PSD-1 and OTS. Failures requiring revision were 23% (18/77) PSD-1, 0% PSD-2, and 3% (1/35) OTS. Most common modes of failure were tibial subsidence (56%) and polyethylene locking mechanism failure (22%) in PSD-1.

**Conclusion:**

Catastrophic failure was seen in the PSD-1 group with tibial subsidence and polyethylene locking mechanism failure. PSD-2 had better early Knee Society function scores, shorter hospital stay, lower hemoglobin drop, radiographic alignment, and no failures compared with PSD-1 and OTS.

Total knee arthroplasty (TKA) is a commonly done, successful surgical treatment to treat end-stage knee osteoarthritis.^1^ With modern surgical techniques and advances in implant technology, primary TKA can result in high 15- to 20-year implant survivorship with some studies reporting good survivorship greater than 20 years.^2,3,4,5^ However, it has been reported that up to one of five patients with TKAs are dissatisfied at 1 year.^6,7,8,9^ Recent research has been focused on improving component alignment, functional outcomes, and patient satisfaction after TKA, and patient-specific design (PSD) TKA was developed to provide patient-matched instrumentations and implants.^1^ Although previous investigations have been somewhat mixed in regard to component alignment, patient satisfaction, and pain scores,^1,2,10^ PSD TKA has been proven to improve accuracy of implantation, reduced surgical time, and facilitate the workflow in the operating room by using fewer surgical instrument.^10,11^

The goal of our study is to retrospectively compare (1) patient-reported outcome scores, (2) radiographic outcomes, and (3) complication/revision rates between PSD TKA versus standard off-the-shelf (OTS) TKAs.

## Methods

Institutional Review Board approval was obtained for a retrospective study from January 2011 until December 2014. The charts of all patients receiving a PSD- or OTS-designed TKA during the study period were reviewed, and they were included in the study if all inclusion criteria were met. The inclusion criteria were patients aged 40 to 85 years, with end-stage degenerative knee osteoarthritis, body mass index (BMI) ≤ 40 kg/m^2^, and medically fit to undergo primary knee replacement. Exclusion criteria included patient's age ≤ 40 or ≥ 85 years, BMI ≥ 40 kg/m^2^, previous known allergy to nickel, active infection, and previous partial knee replacement. All patients were given the opportunity to choose between PSD or OTS after carefully explaining the two options, and patients were allowed to make the final decision regarding the implant used.

The PSD group had TKAs done using patient-specific cutting guides for the femur and tibia together with custom-made femoral and tibial components. Preoperative CT scanning of the entire surgical lower extremity including the hip, knee, and ankle was done at least 6 weeks before surgery, according to a standard scanning protocol designed to calculate the mechanical axis of the leg and to determine sizing of the knee joint. Proprietary software was used by the manufacturer to create virtual 3D models of the tibia and femur, and the program was used to determine the optimal size and shape of the prosthetic tibial and femoral components. Patient-specific disposable cutting guides were made of polyamide and shipped to the operating room together with the custom implants in standard sterile packaging per manufacturer's guidelines. During the study period, the manufacturer of the PSD implant changed the polyethylene locking mechanism and the shape of the polyethylene due to noted early failures thus creating a modified implant. The polyethylene has a lateral component and a medial component. All patients who had the early PSD implants were categorized in the PSD-1 group, and those with the modified implants were in the PSD-2 group. The use of the initial designed PSD implant was stopped after the early failures were noted. The PSD-1 implant was a cruciate-retaining total knee (iTotal G2 system; Conformis), and the PSD-2 implant was a cruciate-retaining total knee (iTotal G2 plus system).

In the OTS group, unilateral TKAs were done using posterior stabilized implants (Genesis II knee; Smith and Nephew). Standard weight-bearing radiographs including AP view of the lower extremities including the hip, knee, and ankle on the same image were used to determine the preoperative mechanical axis and also help determine the amount of valgus cut (3°, 5°, or 7°) to achieve a neutral mechanical axis postoperatively. In this control group, all cuts were done with intramedullary guides for the femur and extramedullary guides for the tibia using standard techniques. Preoperative templating was done to suggest sizes; however, final sizing was done using the standard jigs before the appropriate femoral and tibial components were implanted. All TKAs in the three groups were done during the study period by a single surgeon (T.A.C.) at a single institution, and all components were cemented. Three-peg all-polyethylene patella components were cemented using a free-hand cut, and conventional sizing methods were used in all knees.

Demographic data were collected including age, BMI, and laterality. Preoperative hemoglobin and hemoglobin before discharge were recorded. Blood loss during surgery and the need for blood transfusion during hospitalization were also recorded as well as length of hospital stay. A tourniquet was applied to the surgical extremity before incision and was taken down after application of final dressing. Tranexamic acid was given topically in all cases.

Outcomes data were obtained including knee range of motion and Knee Society scores preoperatively and postoperatively at 3, 6, 12 weeks, 6 months, and final follow-up. Knee Society scores were considered clinically significant if there was a difference of 5.9 points or greater for knee scores among the groups and 6.4 points or greater for function scores among the groups.^12^ Complications were also recorded, and additional surgeries including revision surgery were recorded. All patients were followed for a minimum of 2 years or until implant failure requiring revision surgery.

All patients had preoperatively and postoperative standard weight-bearing radiographs including AP, 45-degree posterior-anterior, lateral, and sunrise views of the surgical extremity capturing the hip and ankle joints on the same image for the AP view. Radiographic parameters including preoperative mechanical axis alignment and postoperative mechanical-axis alignment, tibial slope, femorotibial angle, coronal-tibial angle, and patella tilt were assessed according to previously published methods.^13,14^ Postoperative radiographs were also assessed for any evidence or radiolucency, fractures, loosening, and subsidence at all postoperative visits. Statistical analyses were done using the analysis of variance and Tukey post hoc analysis test.

## Results

### Demographics, Length of Stay, and Hemoglobin Drop

A total of 278 patients (PSD-1 = 94, PSD-2 = 48, OTS = 136) and 308 knees (PSD-1 = 106, PSD-2 55, OTS = 147) were identified for the study, and 136 patients (154 knees) were available at minimum 2-year follow-up. Follow-up was 50% at 2 years. The PSD-1 group had 71 patients (77 knees), the PSD-2 group had 30 patients (36 knees), and the OTS group had 39 patients (41 knees). The average age was 62.7 ± 8.4 years, and mean follow-up was 3.1 ± 1.5 years. The average age per group was 62.5 years (PSD-1), 62.8 years (PSD-2), and 63.0 years (OTS). There was no significant difference (*P* = 0.976) in age between the groups (Table 1). The average BMI for the three groups was 30.3 kg/m^2^ (PSD-1), 28.9 kg/m^2^ (PSD-2), and 34.4 kg/m^2^ (OTS). The overall average BMI for all groups was 30.9 kg/m^2^. The OTS group had a significantly (*P* < 0.01) higher BMI than the other groups (Table 1). The average length of stay for the PSD-2 group (2.1 days) was significantly less than the PSD-1 group (2.9 days, *P* < 0.01) and the OTS group (3.3 days, *P* < 0.01).

**Table 1:** Demographic, Length of Stay, and Hgb Drop Data

	PSD-1	PSD-2	OTS	*P* Value	Comments
Age (yr)	62.65 ± 8.3	62.78 ± 6.7	63.03 ± 10.1	0.976	
BMI (kg/m^2^)	30.34 ± 4.5	28.85 ± 5.2	34.44 ± 7.1	<0.001^a^	PSD-1 versus OTS (*P* < 0.01) PSD-2 versus OTS (*P* < 0.01)
Length of stay (d)	2.88 ± 1.1	2.08 ± 0.6	3.3 ± 1.2	<0.001^a^	PSD-1 versus PSD-2 (*P* < 0.01) PSD-2 versus OTS (*P* < 0.01)
Hgb drop (g/dL)	0.87 ± 0.9	0.61 ± 0.3	1.20 ± 1.3	0.031^a^	PDS-2 versus OTS (*P* < 0.05)

BMI = body mass index, Hgb = hemoglobin, PSD = patient-specific design

Hemoglobin delta between preoperative and postoperative hemoglobin (usually on the day of discharge) in the PSD-2 group (0.61 g/dL) was significantly less (*P* < 0.05) than the OTS group (1.20 g/dL) but not significantly different from the PSD-1 group (0.87 g/dL) (Table 1). Intraoperative blood loss was minimal because of the use of tourniquet for the entirety of all cases together with the application of topical tranexamic acid for all cases. Only one patient in the entire cohort (OTS group) had a blood transfusion (2 units). This patient had a starting hemoglobin of 11.5 g/dL and was admitted to the hospital for 12 days after the TKA surgery due to pulmonary embolism that developed on postoperative day 3 which required anticoagulation therapy with Lovenox and Coumadin. This patient also developed a midcalf hematoma on postoperative day 8 that did not require surgical intervention and a hemoglobin drop to 7.1 g/dL for which 2 units of packed red blood cells were transfused. At discharge, the hemoglobin measured 10 g/dL.

### Knee Society Scores

There was a continuous improvement in the Knee Society knee score at all postoperative time points until final follow-up in all groups. At 3 weeks, the PSD-1 group had clinically significant (*P* < 0.05) higher scores compared with the OTS group, and at 6 weeks, the PSD-2 group had clinically significant (*P* < 0.01) higher scores compared with the OTS group. However, after 6 weeks, there was no difference between any of the groups with regard to the Knee Society knee score. All groups had excellent scores at final follow-up (Table 2).

**Table 2:** Knee Society Knee Score

Timeline	PSD-1	PSD-2	OTS	*P* Value	Comments
Preop	55.5 ± 8.3	54.2 ± 6.7	53.7 ± 10.1	0.720	
3 wk	68.1 ± 13.8	66.3 ± 10.1	60.8 ± 12.7	0.019^a^	PSD-1 versus OTS (*P* < 0.05)
6 wk	76.5 ± 11.6	78.8 ± 9.1	71.1 ± 10.4	0.009^a^	PSD-2 versus OTS (*P* < 0.01)
12 wk	84.3 ± 10.9	85.5 ± 11.3	82.4 ± 7.6	0.484	
6 mo	89.3 ± 8.9	91.3 ± 11.4	89.4 ± 4.4	0.546	
Final	94.6 ± 7.6	95.3 ± 13.3	91.9 ± 11.0	0.377	

PSD = patient-specific design

Excellent = 80 to 100, good = 70 to 79, fair = 60 to 69, and poor = ≤ 60.

There was a continuous improvement in the Knee Society function score at all postoperative time points until final follow-up in all groups. The PSD-2 group had clinically significantly higher scores compared with the PSD-1 group at all postoperative time points up to 6 months (*P* < 0.01). The PSD-2 group also had statistically significantly higher scores compared with the OTS group at 3, 6 weeks, and 6 months (*P* < 0.05); however, these scores were clinically significant only at 3 and 6 weeks. After 6 months, all groups had excellent scores, and there was no difference between the groups (Table 3).

**Table 3:** Knee Society Function Score

Timeline	PSD-1	PSD-2	OTS	*P* Value	Comments
Preop	56.1 ± 7.5	53.2 ± 5.2	48.9 ± 7.1	0.135	
3 wk	57.4 ± 22.4	72.3 ± 9.8	61.2 ± 14.7	<0.001^a^	PSD-1 versus PSD-2 (*P* < 0.01) PSD-2 versus OTS (*P* < 0.05)
6 wk	72.2 ± 15.7	82.2 ± 8.0	73.3 ± 11.2	0.001^a^	PSD-1 versus PSD-2 (*P* < 0.01) PSD-2 versus OTS (*P* < 0.01)
12 wk	82.1 ± 12.5	89.2 ± 5.4	85.0 ± 5.6	0.002^a^	PSD-1 versus PSD-2 (*P* < 0.01)
6 mo	83.9 ± 17.5	93.4 ± 10.6	91.9 ± 3.5	0.001^a^	PSD-1 versus PSD-2 (*P* < 0.01) PSD-1 versus OTS (*P* < 0.05)
Final	91.4 ± 18.6	96.1 ± 11.5	95.1 ± 5.3	0.293	

PSD = patient-specific design

Excellent = 80 to 100, good = 70 to 79, fair = 60 to 69, and poor = ≤ 60.

### Radiographic Outcomes and Final Range of Motion

There was no statistically significant difference between the groups in preoperative (*P* = 0.728) and postoperative (*P* = 0.349) mechanical alignment, as well as final range of motion (0.629). The PSD-2 group showed no statistically notable differences in femorotibial angle, coronal-tibial angle, or tibial slope when compared with normal femorotibial angle (5°), normal coronal-tibial angle (0°), and normal tibial slope (5°). The PSD-1 and OTS groups showed femorotibial angle, coronal-tibial angle, and tibial slope that were statistically different from normal (Table 4). All three groups showed no statistically notable difference in patella sulcus angle when compared with normal of 0°.

**Table 4:** Radiographic Outcomes Including Preoperative Mechanical Axis (MA), Postoperative Mechanical Axis, Femorotibial (Fem-Tib) Angle, Coronal-Tibial (Cor-Tib) Angle, Tibial Slope, and Patella Sulcus (PS) Angle

	PSD-1	PSD-2	OTS	*P* Value
Preop MA	−3.97° ± 3.5°	−3.89° ± 3.46°	−3.32° ± 5.2°	0.728
Postop MA	−1.34° ± 4.6°	−0.35° ± 1.8°	−1.43° ± 2.8°	0.349
Fem-tib angle	4.09° ± 2.7° (*P* = 0.004)^a^	4.10° ± 3.0° (*P* = 0.075)	2.29 ± 3.8° (*P* < 0.001)^a^	
Cor-tib angle	1.08° ± 1.9° (*P* < 0.001)^a^	−0.11° ± 1.0° (*P* = 0.524)	−1.46° ± 1.5° (*P* < 0.001)^a^	
Tibial slope	6.40° ± 2.9° (*P* < 0.001)^a^	5.53° ± 3.9° (*P* = 0.420)	4.00° ± 2.5° (*P* = 0.028)^a^	
PS angle	0.22° ± 1.4° (*P* = 0.143)	0.83° ± 2.8° (*P* = 0.083)	−0.77° ± 2.9° (*P* = 0.153)	
Final ROM	124.2° ± 6.0°	123.8° ± 7.4°	122.7° ± 8.2°	0.629

PSD = patient-specific design, ROM = range of motion

Negative values for MA, Fem-Tib, and Cor-Tib indicate varus alignment, while positive values indicate valgus alignment. Negative values for PS angle indicate medial patella tilt, and positive values indicate lateral patella tilt. ROM data.

### Complications

**Figure 1:**
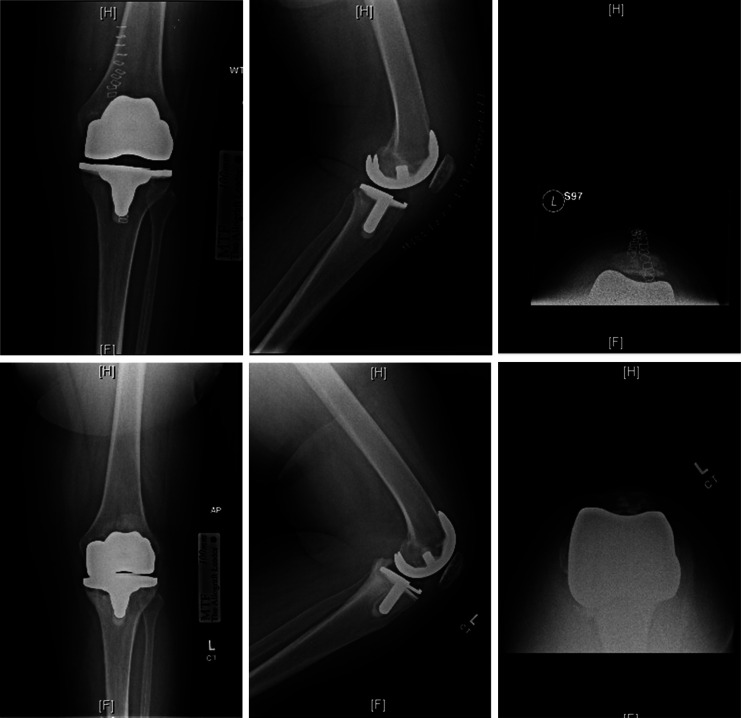
Top three images are immediate postoperative AP, lateral, and sunrise views of the left knee of a 69-year-old woman status-post total knee arthroplasty with PSD-1 implant; bottom three images are 3-year postoperative AP, lateral, and sunrise views of the left knee of the same patient with posterior tibial component subsidence evident on the lateral view.

**Figure 2:**
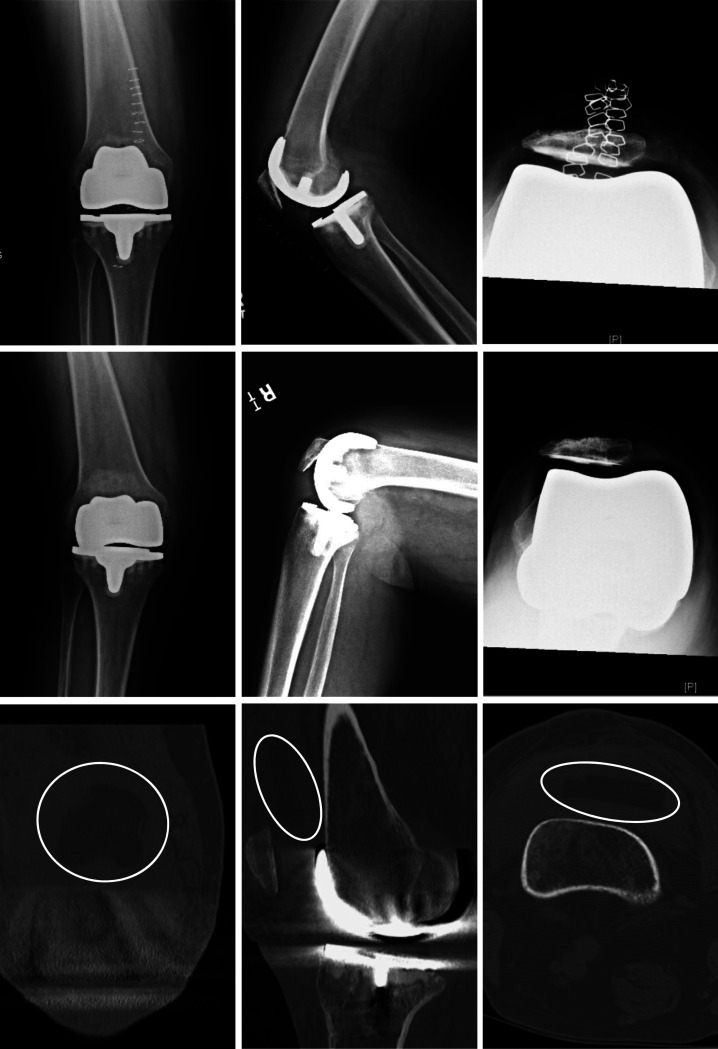
Top three images are immediate postoperative AP, lateral, and sunrise views of the right knee of a 62-year-old woman status-post total knee arthroplasty with PSD-1 implant; middle three images are 6-month postoperative AP, lateral, and sunrise views of the right knee of the same patient with lateral polyethylene dislocation; Bottom three images are coronal, sagittal, and axial CT scan images of right knee showing dislocation of lateral polyethylene insert (encircled) lodged in the suprapatellar recess

There was a 23% (18/77) failure rate in the PSD-1 group requiring revision. The most common modes of failure were tibial subsidence (Figure 1) which accounted for 66% and polyethylene locking mechanism failure (Figure 2) which accounted for 22%. The remaining 12% were treated by outside surgeons, and the reasons for failure were unknown. There were no complications in the PSD-2 group. One patient in the OTS group had patella component loosening that required a revision surgery.

## Discussion

The main findings of this study include the unexpected high early failure rate in the early PSD implant that was attributed to failure of the polyethylene locking mechanism and tibial component subsidence. After the manufacturer changed the design with creation of the modified implant, no failures were seen. The modified PSD-2 implants had better early (up to 6 weeks) Knee Society outcome scores compared with standard OTS implants. Both implant types had excellent postoperative mechanical alignment and ranges of motion. The PSD-2 group showed statistically significantly more normal radiographic alignment compared with the other 2 groups; however, the clinical significance is unknown.

There are several advantages of PSD total knee systems including less surgical instrumentation sets requiring smaller sterile area in operating room, less hospital inventory spaces, and shorter surgical set-up time.^15,16,17,18,19^ Multiple trays are not required, and the presurgical setup is simplified with PSD TKAs when compared with OTS knees that require multiple trays which can result in greater delays in preoperative set up time, postoperative clean up, and turnover time, in addition to the intraoperative search and find time.^16^ Furthermore, several studies have shown reduction in procedural time due to proper sizing of the components (completed preoperatively by the manufacturer using 3D imaging modalities), improved tibial and femoral implant fit, and improved rotational alignment with less mechanical-axis outliers.^1,15,17,19^ The reduction in turnover/surgical time and the lack of the need for autoclaving multiple surgical instrument sets can potentially decrease episodes-of-care costs. With PSD implants, hospitals and surgical center do not have to keep a large inventory of different sized implants, as every implant is made specifically for one patient and shipped directly to the surgical center. Although a CT scan is needed preoperatively for PSD implants, this is mostly covered by insurance companies with a small copay from the patient depending on their insurance plan.

PSD TKA minimizes bone cuts, eliminates the use of intramedullary rods to determine alignment, and provides complete metal coverage of cut bony surfaces which can decrease blood loss due to avoidance of notable intramedullary and cancellous surface bone bleeding.^15,19^ This study showed a statistically significantly lower perioperative blood loss in the PSD-2 group when compared with the OTS group, with one patient in the OTS group requiring blood transfusion (due to pulmonary embolism and midcalf hematoma) compared with none in the PSD groups. However, clinical significance of this difference in blood loss among the groups is unknown. Also, the patients with PSD-2 had decreased length of stay compared with the OTS group or the PSD-1 group, which is consistent with previously reported investigations.^20^ The decreased length of stay is likely due to improved early function and mobility, as it relates to less knee swelling and pain, and other factors that we might be unable to identify. Surgical start times were not dictated by implant choice; they were set by surgical posting and were random. We do not believe the surgical start times had a notable effect on hospital length of stay among the groups. We also report improved short-term functional outcome scores up to 6 weeks. Although the benefits were plateaued after 6 weeks, we believe that this is critical and valuable for the patients during early postoperative recovery phase after TKA. Such benefits may allow the patient to actively participate in a recovery program and allow quicker return to his/her work or desired activities.

The early PSD implant polyethylene had a high posterior lip, and the patient-specific guides only allowed up to a maximum 5° of posterior tibial slope which was the limit set by the FDA. It was theorized that during deep flexion, there was posterior impingement due to limited posterior tibial slope and high posterior lip, and, over time, there was a compensatory posterior subsidence of the tibial baseplate on the tibia. This led to instability and ultimately failure of the polyethylene locking mechanism. The design changes to the early PSD implant included improved locking mechanism of the polyethylene to the tibial baseplate, a reduced posterior lip on the polyethylene, and allowance of more posterior tibial slope. The resultant modified PSD did not show any early failures in this study.

High viscosity cement (Cobalt; DJO surgical) was used for all cases. Kopinski et al^21^ showed evidence of cement debonding from tibial component after total knee using the high viscosity cement (Cobalt). Given that this cement was used for all patients in this study and subsidence was only noted in the PSD-1 group, it is theorized that the cement did not play a role in these failures.

There was a statistically notable difference in the BMI between the PSD and OTS groups with the OTS group having a higher BMI. There have been multiple studies that have evaluated the effects of BMI on clinical outcomes after TKA and have shown that Knee Society score, Western Ontario and McMaster Universities Osteoarthritis Index, Hospital for Special Surgery score, and Visual Analog Scale are not affected by BMI when patients have a BMI < 40 kg/m^2^.^22,23^ Although there were differences in average BMI between the PSD and OTS groups, all patients had to have a BMI < 40 kg/m^2^ to be included in this study.

There are several limitations of this study. First, this study was not randomized or blinded, and it was retrospective. Second, the study compared posterior stabilized TKA (OTS group) with cruciate retaining TKA (PSD groups) with different manufacturers. However, there have been multiple studies that have reported similar outcomes between cruciate-retaining and posterior-stabilized TKAs.^24^ Third, there is only short- to medium-term follow-up.

The strengths of this study include radiographic and clinical evaluation of 136 consecutive patients with sufficient short- to medium-term follow-up by a single surgeon, and this is the first study of its kind to report high catastrophic failure of iTotal G2 system by Conformis, which has been replaced with the modified design (iTotal G2 plus system).

## Conclusion

This study reports an unexpected high incidence of tibial baseplate subsidence, polyethylene locking mechanism, and ultimately failure requiring revision with the early PSD implant which was eliminated with the modified design. The modified designed implant had better early outcome scores only up to 6 weeks, shorter hospital stay, and lower blood loss compared with standard off-the-shelf design TKAs.
